# Influence of Carbon on the Microstructure Evolution and Hardness of Fe–13Cr–xC (x = 0–0.7 wt.%) Stainless Steel

**DOI:** 10.3390/ma14175063

**Published:** 2021-09-04

**Authors:** Michael Harwarth, Adam Brauer, Qiuliang Huang, Mehdi Pourabdoli, Javad Mola

**Affiliations:** 1 Materials Design and Structural Integrity Laboratory, Faculty of Engineering and Computer Sciences, Osnabrück University of Applied Sciences, 49076 Osnabrück, Germany; michael.harwarth@hs-osnabrueck.de (M.H.); adam.brauer@hs-osnabrueck.de (A.B.); 2 Institute of Iron and Steel Technology, Technische Universität Bergakademie Freiberg, 09599 Freiberg, Germany; qiuliang.huang@iec.tu-freiberg.de; 3 Present address: Institute of Energy Process Engineering and Chemical Engineering, Technische Universität Bergakademie Freiberg, 09599 Freiberg, Germany; 4 Department of Materials Engineering, Hamedan University of Technology, Hamedan 65155-579, Iran; mpourabdoli@hut.ac.ir

**Keywords:** stainless steel, phase transformation, thermal analysis, austenite–martensite phase mixtures, hardness

## Abstract

The influence of carbon on the phase transformation behavior of stainless steels with the base chemical composition Fe–13Cr (wt.%), and carbon concentrations in the range of 0–0.7 wt.%, was studied at temperatures between −196 °C and liquidus temperature. Based on differential scanning calorimetry (DSC) measurements, the solidification mode changed from ferritic to ferritic–austenitic as the carbon concentration increased. The DSC results were in fair agreement with the thermodynamic equilibrium calculation results. In contrast to alloys containing nearly 0% C and 0.1% C, alloys containing 0.2–0.7% C exhibited a fully austenitic phase stability range without delta ferrite at high temperatures. Quenching to room temperature (RT) after heat treatment in the austenite range resulted in the partial transformation to martensite. Due to the decrease in the martensite start temperature, the fraction of retained austenite increased with the carbon concentration. The austenite fraction was reduced by cooling to −196 °C. The variation in hardness with carbon concentration for as-quenched steels with martensitic–austenitic microstructures indicated a maximum at intermediate carbon concentrations. Given the steady increase in the tetragonality of martensite at higher carbon concentrations, as confirmed by X-ray diffraction measurements, the variation in hardness with carbon concentration is governed by the amount and stability of austenite.

## 1. Introduction

The addition of 10.5 wt.% chromium to pure iron significantly enhances the corrosion resistance by enabling passivation [1]. Based on their microstructure, stainless steels can be classified into the following five categories: martensitic, ferritic, austenitic, austenitic–ferritic, also referred to as duplex, and precipitation hardenable [2,3]. The room-temperature (RT) microstructure of stainless steels is, on the one hand, controlled by their microstructure at the annealing temperature, and, on the other hand, by the evolution of the high-temperature microstructure upon cooling to RT. For example, a stainless steel with an austenitic microstructure at the solution annealing temperature will either retain its austenitic microstructure or (partially) transform to martensite after cooling to RT, depending on its martensite start (M_s_) temperature [4]. Martensitic stainless steels are obtained if the M_s_ temperature is much higher than RT, such that austenite is fully transformed to martensite before RT is reached [5]. This is the case with martensitic and precipitation hardenable stainless steel grades [6].

Due to its remarkable effects on the solidification mode [7,8,9], the relative stability of austenite [10] and ferrite [11], and the M_s_ temperature [12,13,14], carbon plays an important role in the microstructure evolution of stainless steels. The noticeable effect of carbon on the microstructure formation after welding of stainless steels, for instance, can be implied from its large coefficient in the existing empirical equations for the calculation of the Ni equivalent in Schaeffler diagrams [15]. Furthermore, carbon can influence mechanical properties by interstitial strengthening [16,17]. In the case of austenite-containing stainless steels, such as the steels processed by quenching and partitioning (Q&P), the stability of austenite, and its contribution to mechanical properties, is largely controlled by the solute carbon concentration of the austenitic constituent, which, in turn, regulates its stacking faults energy [18,19] and its response to mechanical loading. Solute carbon can also contribute to the formation of Cottrell atmospheres around dislocations cores, thereby influencing the flow behavior by the static and dynamic strain aging effect [20,21,22,23].

Due to its high affinity for Cr, carbon can also contribute to the formation of Cr-rich carbides, such as M_7_C_3_ and M_23_C_6_ [24,25]. Thermodynamically, the formation of carbides is favored at low temperatures [26]. Kinetically, however, the formation of Cr-rich carbides cannot take place at very low temperatures, where Cr atoms are immobile [27]. The formation of carbides has important consequences for the corrosion and mechanical performance of steels [28]. In view of the corrosion resistance, the formation of Cr-rich carbides is undesirable, as it reduces the solute concentration of Cr, which is crucial to the corrosion performance [3,29]. In contrast, the formation of carbides is desirable in wear-intensive applications, e.g., in razor blades, in tools for plastic molding, or in turbine blades in power plants [30,31,32,33,34]. In the latter case, carbide formation additionally contributes to the wear resistance by raising the M_s_ temperature and ensuring a fully martensitic matrix microstructure without retained austenite [28,35]. 

Given the importance of carbon as an alloying element in stainless steels, especially in martensitic grades, the present work covers a comprehensive study of the effect of carbon on the microstructure evolution of Fe–13Cr–xC stainless steels. In contrast to the existing literature on phase transformations in 13%Cr steels, which deal with a single alloy or a narrow chemical composition range [7,10,36], a wide chemical composition and temperature range is studied in the present work. The microstructure evolution is studied over a broad temperature range, stretching from cryogenic temperatures up to the liquid range. Due to the absence of further alloying elements and a gradual increase in the carbon concentration, with increments of only 0.1 wt.%C, phase transformations shift in a gradual manner. The associated gradual transitions in the phase transformation behavior and transformation temperatures facilitate interpretation of the signals obtained from DSC and dilatometry experiments. The results from experimental characterizations are compared with thermodynamic equilibrium calculations. Furthermore, by adjusting microstructures consisting of different fractions of martensite and austenite at RT, the influence of carbon concentration and phase fraction on hardness is investigated. Finally, the dependence of the lattice parameter on the carbon concentration of martensite and austenite in stainless steels is studied by diffraction analysis. The microstructure–property relationships covered in the present work are especially relevant to the design of processing conditions in novel heat treatment routes, such as Q&P processing.

## 2. Materials and Methods

Fe–13Cr–xC (concentrations are given in wt.%) alloys with x varied between 0 and 0.7 were produced in a Linn High Therm cold-crucible induction melting facility. The Cr and carbon concentrations were quantified by optical emission spectroscopy (Oxford Instruments Analytical Foundry-Master UV, High Wycombe, UK) and combustion analysis (Bruker AXS G4 ICARUS, Karlsruhe, Germany), respectively, and are given in Table 1. The concentration of unintentional elements was low enough to permit neglecting them. To reduce elemental segregation in cast ingots, they were homogenized at 1200 °C for 1 h under a protective Ar gas atmosphere in a Linn HT-1400-G furnace. Afterwards, they were cooled down in an oil bath held at a temperature of 60 °C. 

Materials in the as-homogenized condition were used for thermal analysis by DSC and dilatometry. DSC measurements were conducted in a DSC 404 C Pegasus thermal analyzer. For the measurements, specimens with dimensions of 3.5 × 3.5 × 3 mm^3^ were placed in alumina crucibles. Heating and soaking steps were conducted in vacuum (~10^−4^ mbar). DSC heating and cooling rates were 50 °C/min and 10 °C/min, respectively. To examine the reproducibility of experiments and identify possible artifacts, DSC measurements were performed using two specimens from each condition. Transformation temperatures identified during continuous heating were compared with the thermodynamic equilibrium calculation results based on Thermo-Calc. The TCFE9 database was used for the calculations. Dilatometry measurements using specimens 3 × 3.5 × 10 mm^3^ in size were conducted in a BÄHR-DIL805 dilatometer. Initially, the as-homogenized specimens were heated to 1180 °C in vacuum, which was the upper temperature limit for the dilatometer used. After a soaking time of 1 min to gain a near-equilibrium phase balance, specimens were cooled to RT by blowing Ar into the chamber. The holding time of 1 min is clearly too short to fully eliminate chemical composition inhomogeneities left from the solidification step. Heating and cooling rates in dilatometry cycles were both 20 °C/s. Yang and Bhadeshia have proposed an offset method to identify the apparent M_s_ temperature based on the associated expansion [37]. Their method consists of identifying the temperature at which a predefined fraction of martensite has formed. The offset method requires that the expansion corresponding to the full transformation of austenite be known. The full transformation expansion will be known if transformation proceeds until completion by the time RT is reached. Since this condition was not satisfied for the present alloys with high C concentrations, an alternative method was used. The method for evaluating the results is based on the identification of local minima of dilatometry curves at which the condition d(ΔL/L_0_)/dT = 0 is satisfied [38]. 

Austenitization heat treatments using 10 × 10 × 4.5 mm^3^ specimens were conducted in a muffle furnace. In order to protect the specimens from surface decarburization and oxidation, they were encapsulated in evacuated quartz tubes with an air pressure of less than 2.5 × 10^−3^ mbar. Austenitization heat treatment conditions appropriate for each alloy were selected based on insights from DSC results. A consideration in the design of austenitization conditions was to ensure that Cr-rich carbides were fully dissolved. This was a requirement to obtain as-quenched martensitic–austenitic microstructures in which the chemical composition of each constituent phase corresponded to the average composition. Austenitization conditions applied to the alloys to meet the preceding requirements are summarized in Table 2. Since the quartz tubes containing specimens were charged into a furnace set to target temperature, holding times were only long enough to ensure that the target temperature was reached throughout the specimens. A holding time of nearly 3 min was estimated to be associated with temperature equalization. On this basis, for austenitization temperatures up to 1200 °C (0.1C–0.5C alloys), holding time was set to 10 min. For the austenitization temperature of 1300 °C with a greater risk of surface decarburization (0.6C and 0.7C alloys), holding time was set to 5 min to minimize surface decarburization and maximize usable specimen size. At the end of the austenitization cycle, the specimens were quenched in water at 20 °C (RT). To obtain another series of specimens with higher martensite fractions, a second specimen of each alloy was initially quenched in water at RT and then immediately transferred to a liquid nitrogen tank at −196 °C and held there for 3 min. To determine the evolution of martensite fraction with quench temperature, specimens for magnetic measurements were obtained by quenching to temperatures between 20 °C and −196 °C.

Microstructural examinations were conducted using a Zeiss Axio Imager 2 optical microscope. Specimen preparation for microscopic examinations consisted of mechanical grinding and polishing. The final polishing step was conducted using non-crystallizing colloidal silica suspension as the polishing agent. After proper cleaning, the specimens were etched in Beraha I etching solution for 5 s [39].

In order to determine lattice parameters and phase fractions in as-quenched specimens, X-ray diffraction (XRD) measurements were conducted using a Seifert-FPM URD6 diffractometer with a Co anode (λ = 1.7902 Å). TOPAS software was used for the Rietveld analysis of the XRD patterns. For a more reliable quantification of the paramagnetic austenite phase in specimens, magnetization measurements were conducted using a Metis MSAT device equipped with a Lakeshore 480 fluxmeter. The magnetic field intensity of the device was high enough (>3.77 kOe) to ensure that the ferromagnetic phases (ferrite + martensite) in the bulk of specimens were magnetized nearly until saturation. The equipment returned the ferromagnetic phase content after accounting for the effect of alloying elements through a simple linear calibration procedure, which assigns a demagnetization coefficient to each alloying element contained in the chemical composition. The internal calibration of the device for the demagnetizing effect of Cr was refined manually based on the measured mass magnetization of the 0C alloy as reference with a fully ferritic microstructure. Vickers hardness tests of as-quenched specimens were conducted using a WOLPERT 430 SVD hardness indenter in accordance with the DIN EN ISO 6507-1 standard [40].

## 3. Results and Discussion

### 3.1. Solidification Behavior

**Figure 1:**
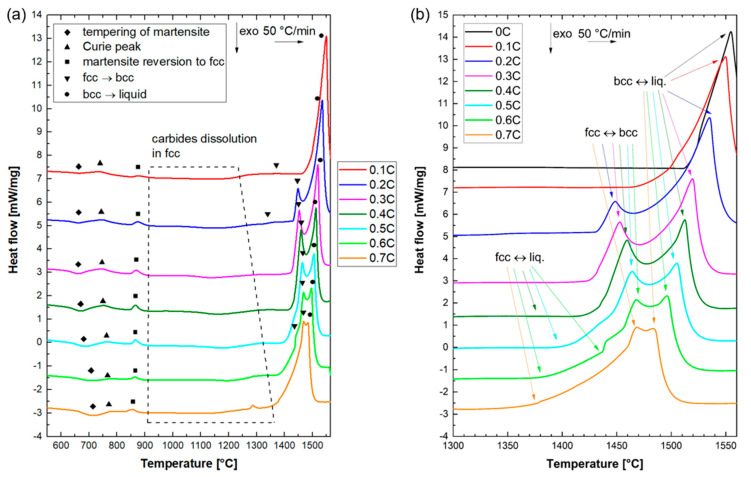
(**a**) DSC results during heating up to 1560 °C; (**b**) an enlarged view of the melting range.

To interpret the sequence of transformations during the heating of alloys, and compare them with the thermodynamic equilibrium calculations, the DSC results during continuous heating were analyzed. The DSC traces of all alloys in the temperature range 550–1560 °C are shown in Figure 1a. Use of the DSC results obtained during heating to the liquid range—instead of those obtained during cooling from the liquid range—offers three considerable advantages. Firstly, there will be no change in the morphology of specimens, from cuboidal to spherical, due to the surface tension effects, which become dominant in the liquid state. A large contact area between the flat faces of the specimens and the crucible will, in turn, ensure a high heat exchange and a strong DSC signal. Secondly, the risk of contamination of the alloys, due to reactions with the surrounding atmosphere, increases in the liquid state. Finally, the possibility of applying a higher heating rate compared to the maximum applicable cooling rate increases the heat flow per unit time, thereby strengthening the acquired signal (peak intensity).

**Figure 2:**
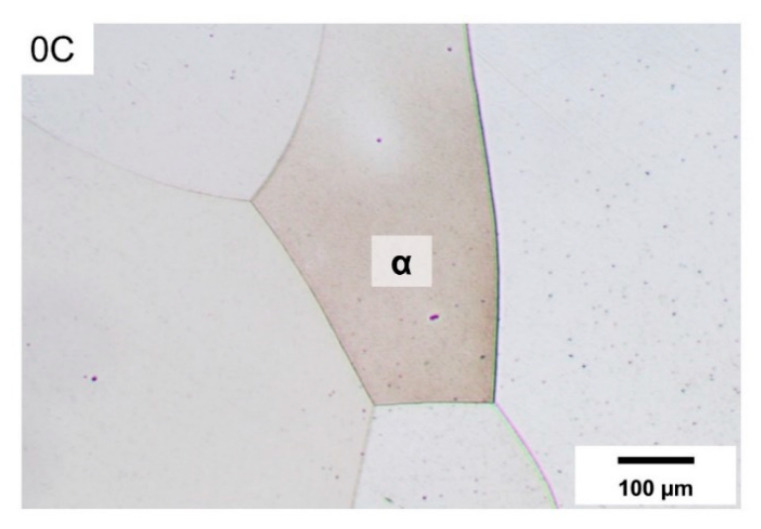
Optical micrograph of the 0C alloy quenched to 20 °C and etched with Beraha I solution.

Within the temperature range shown in Figure 1b, the binary alloy 0C only exhibits a single endothermic peak, starting at nearly 1510 °C. Based on the optical microstructure of the 0C alloy in Figure 2, this alloy exhibits a fully ferritic microstructure at RT. Accordingly, an inspection of the DSC trace at lower temperatures indicated no peaks other than the endothermic peak, due to the ferromagnetic-to-paramagnetic transition of ferrite (Curie temperature peak, T_c_) in the vicinity of 730 °C. In the absence of phase transformations in the solid state, the peak centered about 1550 °C is due to the melting of ferrite and indicates ferritic solidification during the reverse process of cooling from the liquid range.

By adding 0.1% C to the binary alloy, the austenite range expanded and it became possible to obtain a noticeable fraction of martensite at RT. The latter was confirmed by the microstructural examination of as-quenched specimens, as discussed in Section 3.3. Accordingly, the weak exothermic peaks, marked by diamond symbols in Figure 1a (near 650 °C), can be attributed to the stage IV tempering of the pre-existing martensite, namely, the formation of Cr-rich carbides [41]. This pre-existing martensite reverses to austenite at higher temperatures, giving rise to the endothermic peak observed near 850 °C (square symbols in Figure 1a). The peaks due to the tempering and reversion of martensite were separated by the Curie peak. At still higher temperatures, the stability of ferrite increased at the expense of austenite, thereby leading to a gradual increase in the fraction of ferrite. The broad endothermic peak in the 0.1C alloy, in the approximate temperature range 1180–1410 °C (Figure 1a), can be explained by a gradual increase in the fraction of delta ferrite. A fully ferritic microstructure is obtained in the temperature range of 1410–1470 °C. The large endothermic peak emerging at nearly 1470 °C can be ascribed to the incipient melting of ferrite. Similar to the 0C alloy, a ferritic solidification mode is implied for the 0.1C alloy.

Microstructural examinations of alloys containing C contents that are equal to or greater than 0.2% in the as-quenched condition (Section 3.4) indicated that they were capable of developing a fully austenitic matrix microstructure at high temperatures. Due to the high hardenability of alloys, austenite can readily transform to martensite during cooling from the prior homogenization step. Accordingly, the DSC traces of the alloys 0.2C–0.7C exhibited an exothermic peak, due to the tempering of pre-existing martensite in the as-homogenized condition and its endothermic reversion to austenite at temperatures typically of the order of 850 °C. The occurrence of these two reactions has also been demonstrated by continuous dilatometry heating cycles of steels with chemical compositions resembling that of the 0.5C alloy in the present work [42]. Conducting a soft annealing heat treatment associated with the formation of ferrite and carbides, prior to DSC measurements, would eliminate the tempering peak.

In the high-temperature range relevant to melting, at least two overlapping endothermic peaks can be identified in the DSC traces of 0.2C–0.7C alloys. These peaks are attributed to the formation of ferrite (triangles pointing downwards in Figure 1a) and liquid (circles in Figure 1a). The peak due to the formation of liquid was broader than the ferrite formation peak, and exhibited a maximum at a higher temperature. Depending on the carbon concentration, melting started either by the direct liquification of austenite (alloys 0.4C–0.7C) or by liquid formation, subsequent to the partial formation of ferrite (0.2C and 0.3C alloy). In the latter case, the partitioning of carbon between austenite and ferrite, and the changes in the relative stability of austenite and ferrite relative to the liquid phase, is expected to have influenced the onset of melting in each phase.

For the 0.2C alloy, austenite starts to gradually transform into ferrite at nearly 1330 °C, which is accompanied by the enrichment of carbon in the austenite phase, as ferrite can only dissolve up to 0.1%C. As a result, the austenite, which starts to melt at nearly 1430 °C, contains more than the nominal carbon content of 0.2%. The formation of the liquid phase from austenite, and the associated carbon depletion of austenite, increases the thermodynamic driving force for its transformation to ferrite. For instance, the thermodynamic equilibrium calculation results by Thermo-Calc indicate that the temperature associated with the formation of delta ferrite from austenite increases from 1044 °C to 1168 °C as the C concentration is increased from 0.1% to 0.2%. As a result, the second ferrite formation peak in the 0.2C alloy emerges shortly after liquid formation begins. Since liquid and ferrite phases form jointly from austenite above 1430 °C, the peritectic reaction in reverse (fcc → bcc + liquid) is implied.

In contrast to the 0.2C alloy, no peak attributable to the solid-state formation of ferrite from austenite was observed in the 0.3C alloy. At about 1425 °C, austenite starts to melt. Due to the absence of ferrite, the carbon content of austenite corresponds to the nominal carbon content. Liquid formation and the associated carbon depletion of the surrounding austenite regions in turn trigger the formation of ferrite. Accordingly, the onset of liquid formation and ferrite formation from austenite in the 0.3C alloy coincide, namely, DSC peaks for both reactions emerge simultaneously (reverse peritectic reaction). 

In the case of the 0.4C–0.7C alloys, melting begins in the austenite phase, without any prior ferrite formation. For the 0.4C alloy, the melting of austenite begins at about 1415 °C, resulting in a decrease in its carbon concentration and its destabilization with respect to ferrite. Therefore, starting at about 1435 °C, melting and ferrite formation occur in parallel. This is similar to the behavior observed for the 0.3C alloy.

Direct melting of austenite in 0.5C, 0.6C, and 0.7C alloys begins at about 1397 °C, 1377 °C, and 1347 °C, respectively. This indicates a decrease in the melting temperature of austenite with an increase in the carbon concentration. The observation of an overlapping endothermic peak during the melting of austenite in these steels indicates the formation of ferrite. Therefore, in spite of the presence of up to about 0.7%C in alloys, austenite becomes destabilized with respect to ferrite, prior to full melting.

In the case of the 0.6C alloy, the formation of ferrite is associated with two peaks (two triangles in Figure 1a). A possible explanation for the occurrence of ferrite in two distinct stages could be its formation by two different mechanisms, e.g., by direct formation from austenite (fcc → bcc) and as one of the products of an inverse peritectic reaction (fcc → bcc + liquid). To a lesser extent, two-stage ferrite formation can also be implied from the DSC curve for the 0.5C steel.

Based on the sequence of transformations during heating into the liquid range, the following implications can be made with regard to the melting process in reverse, namely, solidification. In all alloys, solidification begins with the formation of ferrite. For the 0C and 0.1C alloys, solidification completes without austenite formation, namely, a single-phase ferritic (F) solidification mode [43]. For the 0.2C–0.7C alloys, on the other hand, some austenite is formed in the solidification range, namely, a ferritic–austenitic (FA) solidification mode [44]. Austenite can form directly from liquid (liquid → fcc) or ferrite (bcc → fcc), or from both of them via a peritectic reaction (liquid + bcc → fcc). In the case of 0.4C–0.7C alloys, the final stage of solidification only involves the formation of austenite, namely, liquid → fcc or liquid + bcc → fcc. 

**Figure 3:**
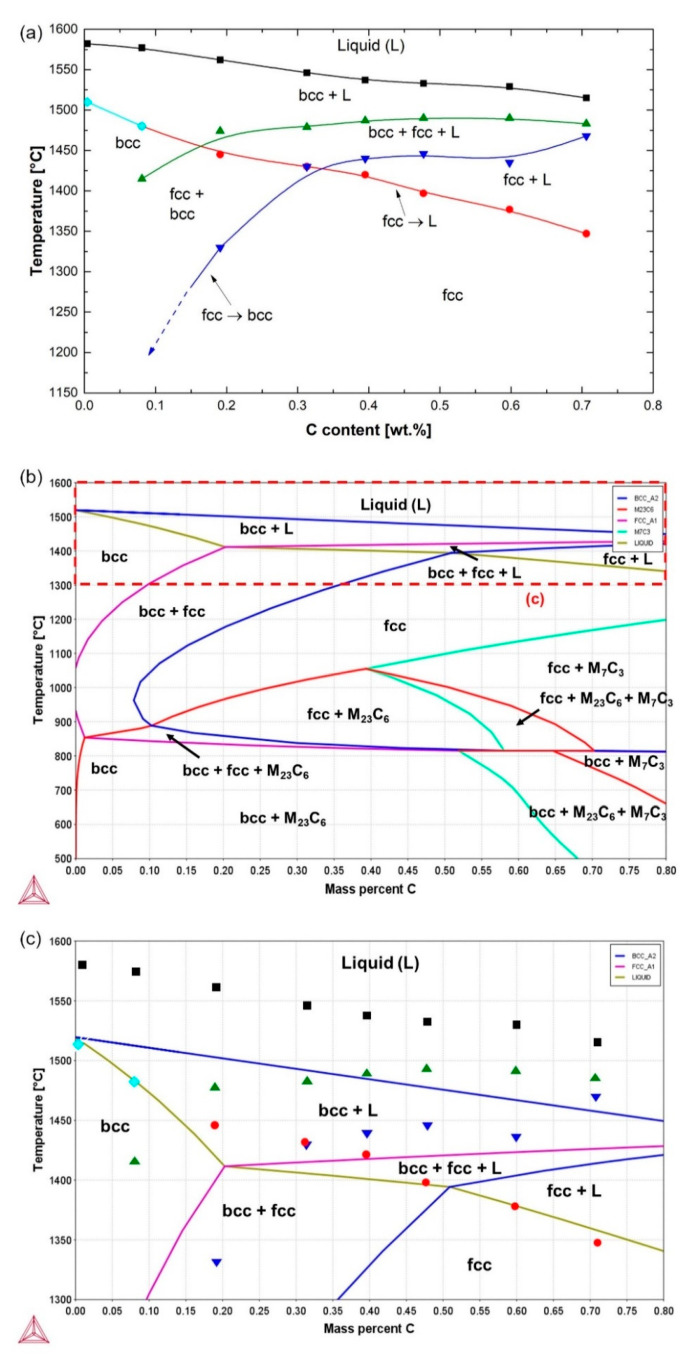
Comparison of the phase transformation temperatures determined based on the DSC signal during continuous heating (**a**) and the results calculated with Thermo-Calc (**b**). (**c**) shows a magnified view of the area marked by a dashed rectangle in (**b**). The DSC-based transformation temperatures marked in (**a**) are superimposed in (**c**).

Figure 3a summarizes the approximate transformation temperatures inferred from the two DSC measurements for each alloy. The points in Figure 3a represent the onset and end of the transformation peaks, namely, where they start to erupt and where they merge with the baseline, respectively. Because of the asymmetrical shape of the peaks, they were determined manually. Error in the identification of the points marked in Figure 3 is estimated to be typically of the order of ±5 °C. In the case of overlapping reactions, error is estimated to increase up to ±15 °C. Due to the fact that these transformation temperatures are obtained during continuous DSC heating with a limited diffusion time in solid phases, they are expected to overestimate equilibrium transformation temperatures [45,46]. Exaggerated transformation temperatures during DSC measurements at relatively high heating rates are also expected if a temperature gradient develops through the thickness of the bottom wall of the DSC crucible, which separates the thermocouple and the specimen.

For a comparison with the DSC results, a pseudo-binary phase diagram for the Fe–13Cr–(0–0.7)C alloy system was calculated by Thermo-Calc (Figure 3b). Given the overestimation of transformation temperatures based on the DSC measurements, the comparison is only qualitative. An enlarged view of the region marked by a dashed rectangle in Figure 3b is provided in Figure 3c. In terms of the solidification mode and the sequence of transformations at high temperatures, the calculated results show a fair agreement with the DSC results. Nevertheless, the critical carbon concentration to obtain a mixture of austenite and liquid in the latest stages of solidification is lower in DSC measurements (~0.35 wt.%) than in thermodynamic calculations (~0.51 wt.%). Furthermore, noticeable differences are observed with regard to the solidification temperature interval, in the sense that the mushy zone based on the DSC measurements is broader. This difference is the most obvious for the 0C steel, for which the experimental results indicate a solidification interval of 70 °C compared to the Thermo-Calc prediction of only 1 °C. On the one hand, inaccuracies might be related to error in the thermodynamic calculation results arising from a weak database calibration. On the other hand, experimental factors, such as the presence of trace elements, dynamic changes in the heat transfer due to surface oxidation, and gradual balling up of specimens after the onset of melting, might have broadened the mushy zone [47]. Pre-existing Cr concentration inhomogeneities arising from the solidification step are another likely contributor to the broadening of the mushy zone. 

Rough estimates of the original Cr segregation after the solidification of the alloys were obtained from thermodynamic and kinetic simulations. The Scheil module of Thermo-Calc is an implementation of the Scheil–Gulliver model for solidification segregation [48,49], which assumes that diffusion occurs infinitely fast within the liquid phase, but there is no diffusion in the solid phases that occur from the liquid. The analytical Scheil equation is commonly derived for an idealized system in which the liquidus and solidus are linear with respect to the composition. During the present simulations, the fast-diffusing interstitial element carbon was allowed to back-diffuse and establish a partial equilibrium [50]. For Cr, on the other hand, equilibrium was established only between the newly formed solid phase and the coexisting liquid, while the solid phase that formed at higher temperatures was regarded as incapable of back-diffusion, namely, it was “frozen”.

**Figure 4:**
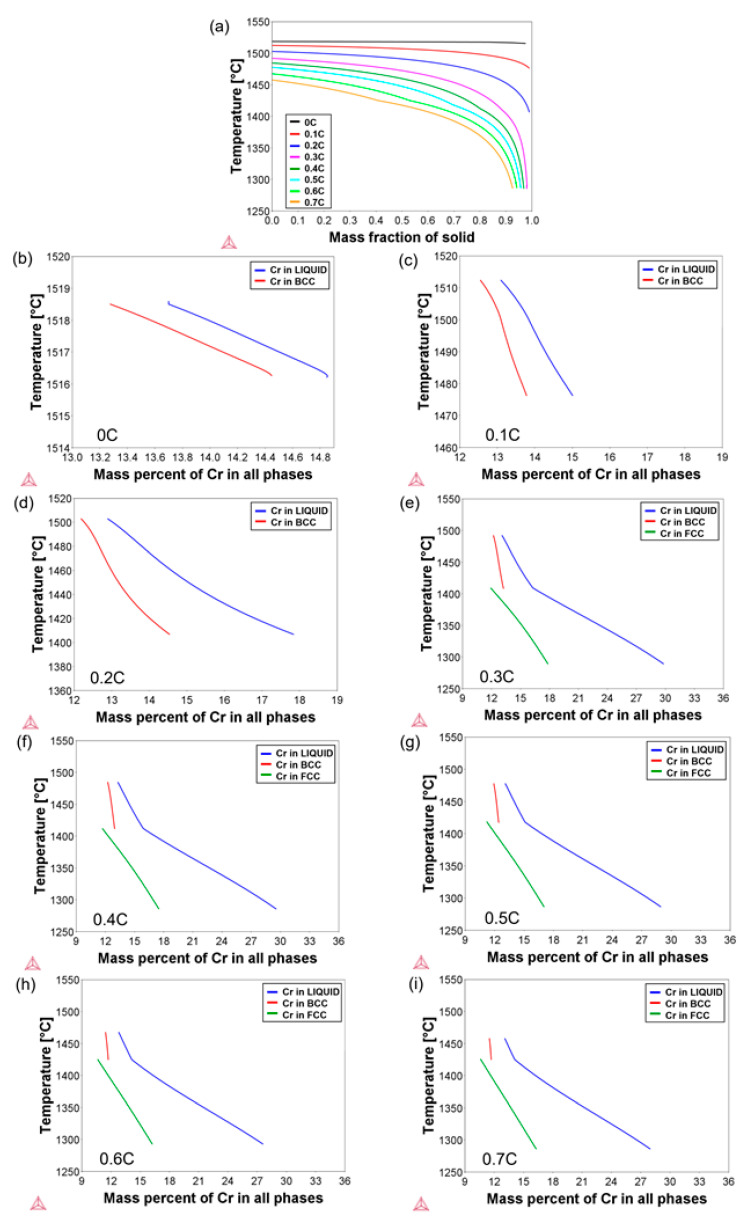
(**a**) Scheil-predicted solidification ranges of the steels; (**b**–**i**) calculated concentrations of Cr in liquid, ferrite, and austenite phases as a function of temperature during solidification of alloys. Simulations were terminated after reaching a threshold liquid phase fraction of 1%.

Figure 4a shows the amount of solid phase formed vs. solidification temperature obtained from the Scheil simulation. It represents the progression of the non-equilibrium solidification temperature range for a cooling rate of 10 °C/s. The solidification range based on the Scheil simulation results for the 0C and 0.1C steels is relatively narrow and agrees well with the thermodynamic equilibrium calculation results. Already for the 0.2C steel, residues of the liquid phase persist at temperatures as low as 1406 °C. This corresponds to nearly 5 °C below the solidus temperature, based on the equilibrium results shown in Figure 3b, and 40 °C below the solidus temperature implied from the DSC-based results presented in Figure 3a. For the rest of the alloys, solidification is complete only at temperatures below 1300 °C, significantly lower than the solidus temperatures suggested by the equilibrium calculations and DSC results. The results clearly demonstrate how the non-equilibrium solidification (absence of back-diffusion) would broaden the mushy zone in the present alloy system. Partial back-diffusion during solidification, or partial homogenization by subsequent thermal treatments, would tend to make the mushy zone narrower in an approach towards equilibrium.

For an estimate of the extent of Cr segregation during solidification, the Scheil module of Thermo-Calc was used. Figure 4b–i show the evolution of the instantaneous Cr concentration in phases during the solidification of all alloys. The results were obtained by applying a cooling rate of 10 °C/s and corresponding to the solidification temperature ranges given in Figure 4a. For each alloy, the Cr concentration of liquid at the respective liquidus temperature indicates its average Cr concentration. Since both ferrite and austenite phases form with a lower Cr concentration than the coexisting liquid, the Cr concentration of liquid constantly increases during the solidification. In the case of the 0.3C–0.7C alloys, the solidification mode is ferritic–austenitic according to Thermo-Calc calculations. Given the lower solubility of Cr in austenite than in ferrite, the Cr enrichment of liquid accelerates in the case of austenite formation. The gradual Cr enrichment of liquid in turn leads to an enrichment of interdendritic regions with respect to Cr. If the last residues of liquid, which are frozen into solid without much change in the chemical composition, are neglected, the segregation ratio for Cr can be calculated based on the maximum and minimum Cr concentrations in solid phases, $\frac{C_{Cr,\;max}}{C_{Cr,min}}$. In the present case, a threshold liquid fraction of 1% was used for the calculation of segregation ratios. The Cr concentrations in both ferrite and austenite phases were considered. Segregation ratios in the range of 1.09–1.54 were obtained in the present alloys. These values are not as large, for example, as the values reported by Kato et al. [51] or Kozeschnik [50], where segregation ratios up to 5 or 2.5 were obtained for Fe–1.5Cr–1C and Fe–1.5Cr–0.7C steels, respectively. Due to the homogenization of alloys prior to DSC measurements, chemical composition inhomogeneities in the specimens used for the DSC measurements are expected to be significantly lower than those based on the present Scheil calculations.

### 3.2. Dissolution of Carbides

As discussed in Section 3.1, DSC measurements were primarily used to interpret the solidification behavior based on the sequence of reactions during melting. In general, methods of thermal analysis, such as DSC and differential thermal analysis (DTA), can also be used for the study of the formation and dissolution of precipitates [52,53], especially if the heat exchange rate is amplified by a high heating rate [54]. In the present case, depending on the carbon concentration and the stability of carbides, carbides might have been present at the homogenization temperature of 1200 °C. Due to the application of oil quenching after homogenization, the possibility of precipitation during cooling can be excluded. Given that austenite partially transforms to martensite during oil quenching, as discussed in detail in Section 3.3, the formation of carbides within the newly formed carbon-supersaturated martensite can take place during the subsequent DSC heating [41,42,55]. Accordingly, exothermic peaks, due to the formation of Cr-rich carbides in martensite, were detected in 0.1C–0.7C alloys (diamond symbols in Figure 1a). The rate of carbide formation in the austenitic regions of the microstructure is comparatively slow [42].

Because of the raised solubility of carbon at high temperatures, carbides preexisting at the homogenization temperature, or those formed in martensite during DSC heating, will dissolve at sufficiently high temperatures. The high temperatures achievable by DSC enable the dissolution temperature range of Cr-rich carbides to be studied, even very stable carbides persisting at temperatures close to the solidus temperature [56]. For 0.1C–0.7C alloys, a broad endothermic peak (peak I) occurs subsequently to the endothermic peak, due to the martensite reversion to austenite. Based on its range of occurrence and its shift to higher temperatures with an increase in the carbon concentration, this peak can be attributed to the dissolution of carbides. Dilatometry measurements using an Fe–13.1Cr–0.47C stainless steel with an austenitic–martensitic microstructure have confirmed that the dissolution of carbides begins immediately after martensite reversion to austenite [39]. Similar observations have been made for high carbon austenitic–martensitic medium Mn steels [57], and can be justified by the high solubility of carbon in austenite compared to that in martensite.

**Figure 5:**
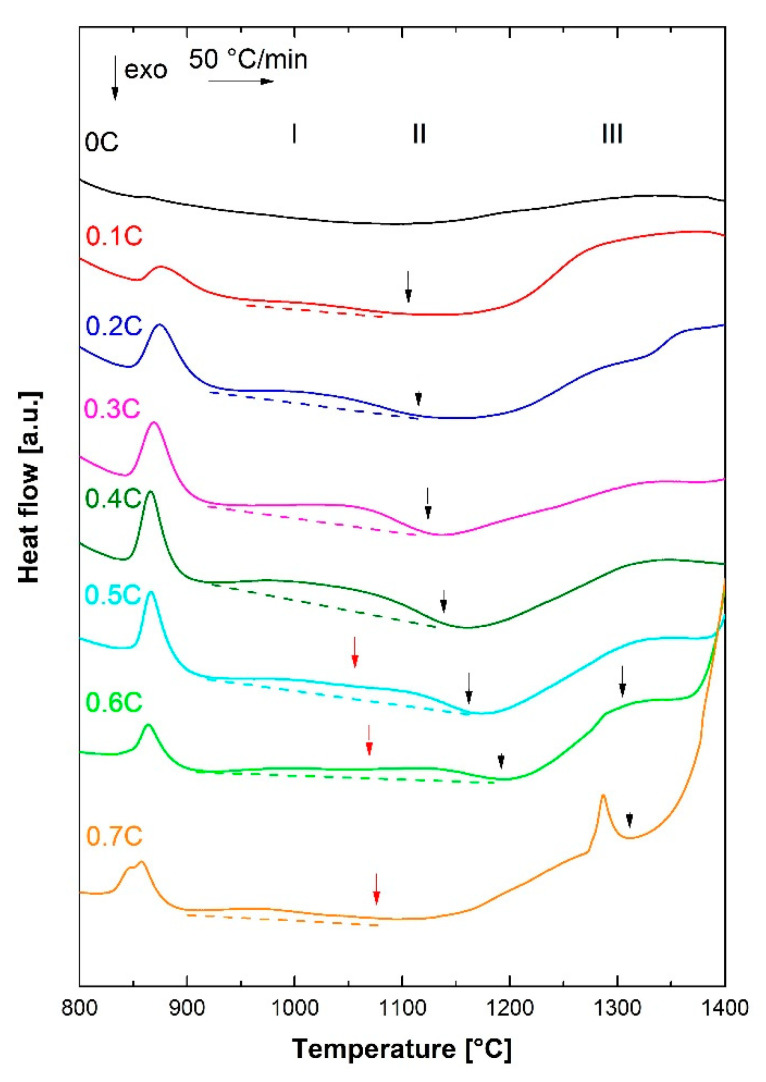
DSC results during heating for the interpretation of the precipitation reactions. For the meaning of arrows, see body text.

In spite of the inaccuracies arising from the identification of the baseline, effort was made to identify temperatures associated with the full dissolution of carbides. The black arrows in Figure 5 mark the approximate end of the endothermic peaks, namely, temperatures associated with the full dissolution of carbides. For the steels 0.1C–0.5C, only one broad endothermic peak could be identified, which is expected to be related to the dissolution of M_23_C_6_ as the most dominant carbide in martensitic stainless steels such as the AISI 420 grade [42]. The thermodynamic stability of M_23_C_6_ can also be implied from the equilibrium phase diagram in Figure 3b. In the case of the 0.5C alloy, the broad endothermic peak shows a mild discontinuity, which is marked by a red arrow in Figure 5. Such a discontinuity is also evident in the case of the 0.6C alloy and might even exist in the DSC curve of the 0.7C alloy, at the temperature marked by a red arrow, but have remained unnoticed due to the particular progression of the baseline. The discontinuity is expected to be related to a change in the type of carbides, for instance, M_23_C_6_ to M_7_C_3_ transformation [26], as also implied from the equilibrium phase diagram at high carbon concentrations. In the case of the 0.6C alloy, another weak endothermic peak emerges shortly below 1300 °C and ends at the temperature marked by a second black arrow. By analogy with [56], this peak is thought to be related to the dissolution of M_7_C_3_ carbides, especially carbides that might have been present and coarsened at the homogenization temperature of 1200 °C. This interpretation is supported by the higher intensity of the latter peak in the case of the 0.7C alloy, whose end marks the full dissolution of all types of carbides.

Compared to the thermodynamic equilibrium calculations, the dissolution temperature of Cr-rich carbides in DSC measurements is somewhat higher. Although the mismatch might be related, to some extent, to the application of a relatively high heating rate during DSC measurements [58,59,60], an underestimation of the dissolution temperature in thermodynamic calculations cannot be excluded. Accordingly, the solution annealing temperatures applied to high interstitial stainless steels often exceed those based on thermodynamic equilibrium predictions [36,61]. In the particular case of the 0.1C alloy with a duplex high-temperature microstructure, C will partition between the coexisting ferrite and austenite phases. Under these circumstances, the carbon concentration of austenite will be higher than the average carbon concentration of the alloy. An increase in the solute C content of austenite in turn increases the dissolution temperature of M_23_C_6_ carbides.

### 3.3. Martensitic Transformation

**Figure 6:**
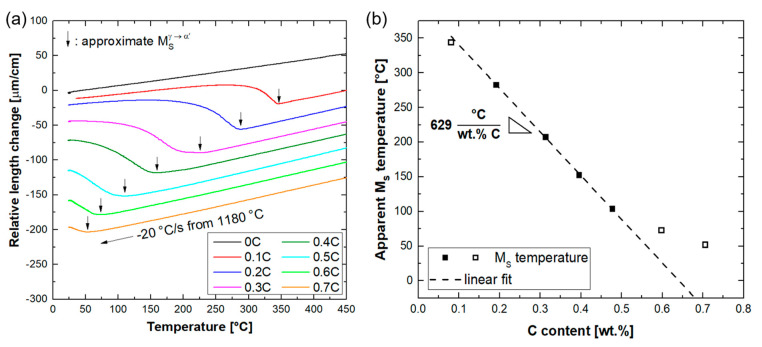
(**a**) Changes in the length of dilatometry specimens quenched from 1180 °C to RT. Except for the 0C alloy, all alloys exhibit remarkable expansions due to martensite formation. The onsets of expansions (M_s_ temperatures) are marked by vertical arrows. For clarity, curves were translated vertically; (**b**) dependence of M_s_ temperature on carbon content. Due to the presence of ferrite in the 0.1C alloy and carbides in 0.6C and 0.7C alloys (open symbols), they were excluded during linear fitting.

Dilatometry measurements were conducted to determine the M_s_ temperatures. By neglecting the differences in the Cr concentration of alloys, it will be possible to determine the carbon concentration dependence of M_s_ temperature in the current alloy system and make a comparison with the literature data. For this purpose, dilatometry specimens were cooled from 1180 °C at a rate of 20 °C/s. The latter temperature was the upper temperature limit of the dilatometer used for the present dilatometry experiments. Relative length changes during the cooling segment are shown in Figure 6a. Except for the austenite-free 0C alloy, all the alloys exhibited a remarkable deviation from near-linear thermal contraction at temperatures below 400 °C. The M_s_ temperatures, determined by the method described in Section 2, are marked by arrows in Figure 6a.

For Fe–13Cr steels with carbon concentrations similar to the 0.5C alloy of the present work, the full dissolution of carbides has been shown to occur at temperatures up to 1180 °C [34,42,62]. For alloys with higher carbon concentrations, the DSC results indicated that carbides persist in the microstructure at 1180 °C. Due to the associated reduction in the solute Cr and C content of these alloys, the M_s_ temperatures quantified for 0.6C and 0.7C alloys by dilatometry are overestimated. Accordingly, they were excluded during the derivation of the relationship between the C content and M_s_ temperature. Based on the linear regression analysis of M_s_ temperatures for 0.2C–0.5C alloys with fully austenitic microstructures at 1180 °C, the M_s_ temperature decreases by about 629 °C per wt.% C (Figure 6b). This coefficient is larger than the coefficient of 423 °C (per wt.% C), based on empirical relationships, to predict the M_s_ temperature in low-alloy steels [63,64]. This deviation indicates the effects of base chemical composition and C–Cr interactions in the present alloy system. The coefficient derived in the present work is also larger than the value of 510 °C (per wt.% C) proposed for Fe–12Cr steels [5]. In the latter case, however, only steels with carbon concentrations up to 0.3% were considered for linear fitting.

### 3.4. As-Quenched Condition

Insights from DSC measurements were used to obtain two-phase austenitic–martensitic microstructures, free of carbides and ferrite. For this purpose, the austenitization temperatures applied to the alloys were higher than the full dissolution temperatures marked by black arrows in Figure 5. The choice of annealing temperatures slightly lower than the dissolution temperatures marked in Figure 5, for instance, in the case of the 0.2C and 0.7C alloys, can be justified by the discussed overestimation inherent to the DSC measurements and the resumed dissolution of carbides during the holding time at each austenitization temperature.

**Figure 7:**
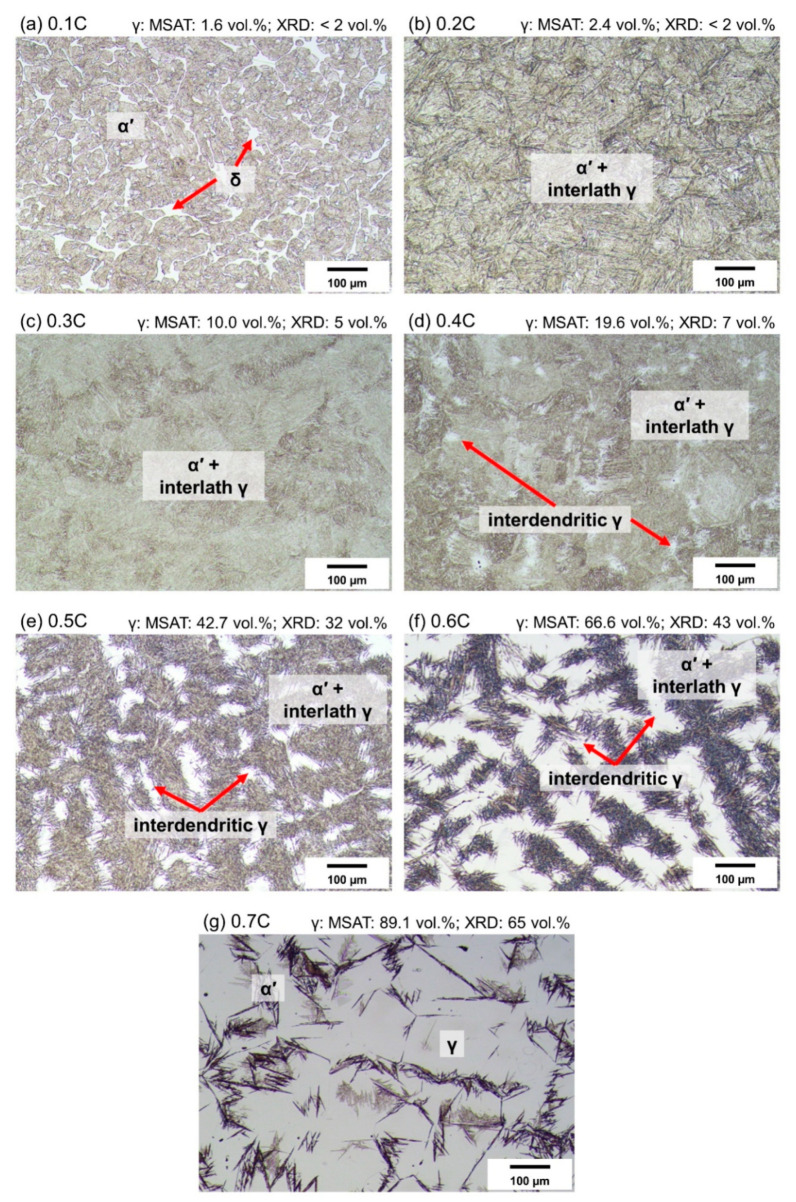
(**a**–**g**) Optical micrographs of 0.1C–0.7C alloys quenched to RT and etched with Beraha I solution. Numbers above optical micrographs denote phase fractions based on magnetic (MSAT) and XRD measurements, respectively.

The microstructures obtained after quenching alloys to RT are shown in Figure 7. The microstructure of the 0.1C alloy consists of martensite and a small fraction of ferrite. The spatial distribution of ferrite resembles that of interdendritic regions in cast microstructures [65]. This observation can be justified by the higher ferrite potential of interdendritic regions, due to the segregation of Cr out of dendrites in the solidification step [21,66]. Due to the high M_s_ temperature of the 0.1C alloy (Figure 7a), the high-temperature austenite is expected to have fully transformed to martensite. This was confirmed by magnetic and XRD measurements (Figure 7a). An increase in the carbon concentration, to 0.2%, resulted in the elimination of ferrite at the austenitization temperature. The decrease in M_s_ temperature compared to the 0.1C alloy increased the likelihood of austenite retention at RT. The austenite fractions quantified by magnetic and XRD measurements were 2.4 vol.% and less than 2 vol.%, respectively. According to magnetic and XRD measurements, the fraction of interlath austenite is higher for the 0.3C alloy. Nevertheless, due to the fine size of the interlath austenite, it could not be identified by light microscopy. In the case of the 0.4C alloy, retained austenite in the form of relatively large islands could be identified by light optical microscopy (Figure 7d). One of the consequences of Cr segregation to interdendritic regions is a reduction in M_s_ temperature. On this basis, the distribution of austenite islands must correspond to the prior interdendritic regions. Nearly 19.6 vol.% of retained austenite was quantified by magnetic measurements. According to XRD measurements, on the other hand, the fraction of retained austenite was of the order of only 7 vol.%. 

Austenite fractions in the as-quenched condition gradually increased with an increase in the carbon concentration. In the case of the 0.6C steel, the martensitic regions highlight the dendritic regions with a lower chromium concentration, hence a higher M_s_ temperature, compared to the interdendritic regions. An increase in the carbon concentration of alloys was also associated with a change in the morphology of martensite from fine laths at low carbon concentrations to coarse plates at high carbon concentrations.

As the numbers marked in Figure 7 indicate, the austenite fractions based on the magnetic measurements are consistently higher than the XRD-based values. The low mechanical stability of austenite in steels with M_s_ temperatures near or above RT implies that some of the austenite in the present steels has transformed to martensite during specimen preparation for XRD measurements [61,67]. Figure 7g (0.7C alloy) clearly shows the formation of mechanically induced martensite (light-brown contrast) next to regions of thermally induced martensite (dark-brown contrast). In addition to the problem of surface sensitivity [68], XRD-based quantifications suffer from a high sensitivity to texture [68,69]. Texture sensitivity is especially relevant to the coarse-grained austenite in the present steels.

**Figure 8:**
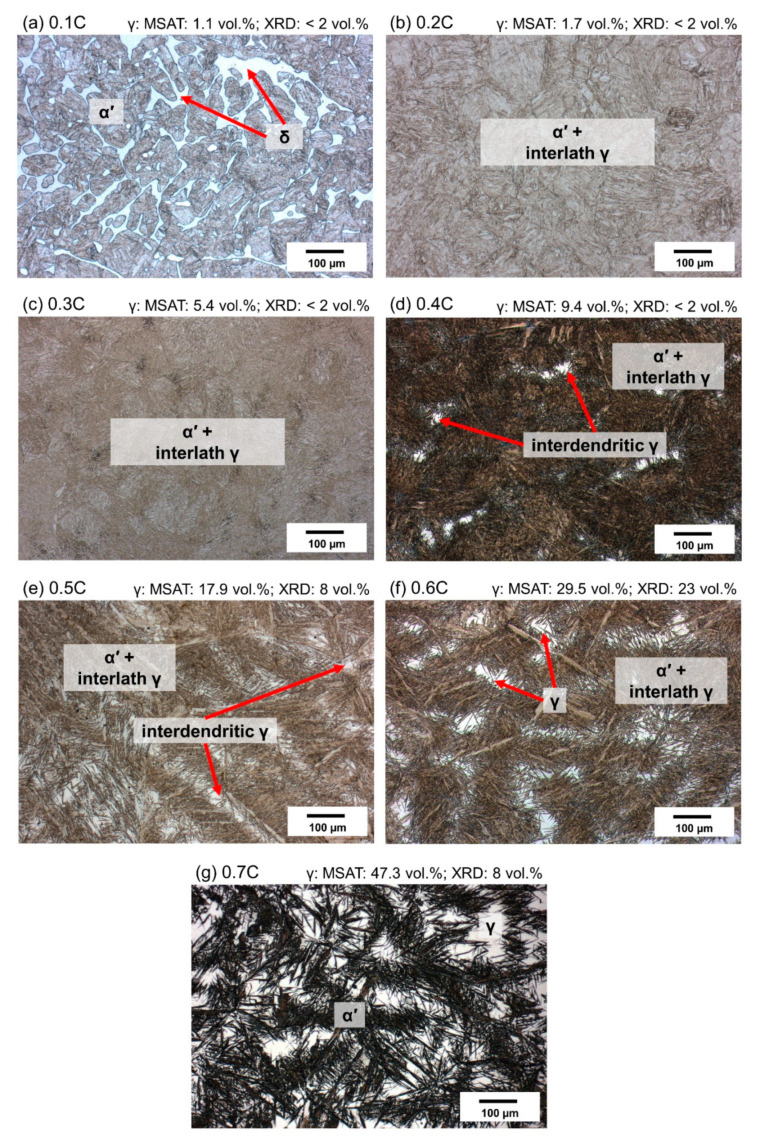
(**a**–**g**) Optical micrographs of 0.1C–0.7C alloys quenched to −196 °C and etched with Beraha I solution. Numbers above optical micrographs denote phase fractions based on magnetic and XRD measurements, respectively.

**Figure 9:**
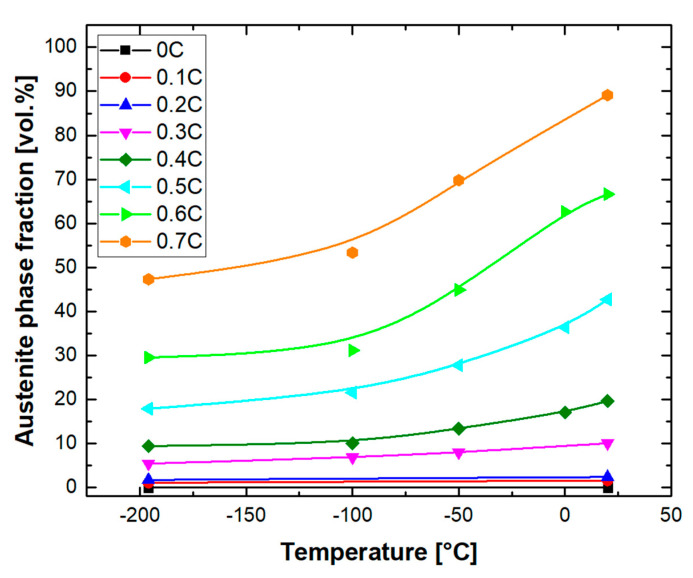
Austenite phase fraction as a function of quench temperature determined by MSAT measurements.

Figure 8 shows optical micrographs of alloys after quenching to −196 °C. According to the microstructures, martensite fractions are higher than those after quenching to 20 °C [70]. For the 0.1C alloy (Figure 8a), the microstructure consists of martensite and δ-ferrite, which resembles that observed after quenching to 20 °C (Figure 7a). For the 0.2C alloy as well, the difference between the austenite fraction obtained after quenching to 20 °C (Figure 7b) and −196 °C (Figure 8b) remained negligible. For the rest of the alloys, an increase in the martensite fraction after quenching to −196 °C was noticeable. Figure 9 summarizes the evolution of the austenite fraction in all the alloys after quenching to various temperatures between 20 °C and −196 °C. The reduction in the austenite fraction at lower temperatures can be explained by the increase in the thermodynamic driving force for martensite formation [36,71,72,73]. Koistinen and Marburger [74] have proposed an empirical equation to describe the non-linear increase in the martensite fraction at temperatures below the M_s_ temperature. The curves in Figure 9 flatten at cryogenic temperatures, implying a more sluggish increase in the martensite fraction. The stabilization of austenite at cryogenic temperatures has been justified by the decrease in the thermodynamic driving force for the martensite formation in the vicinity of the Néel temperature [36,72]. To restrict the thermal stabilization of austenite [75,76], due to holding at 20 °C, quenching to target temperatures below RT was conducted immediately after quenching in water at 20 °C.

### 3.5. Lattice Parameters for Martensite and Austenite

**Figure 10:**
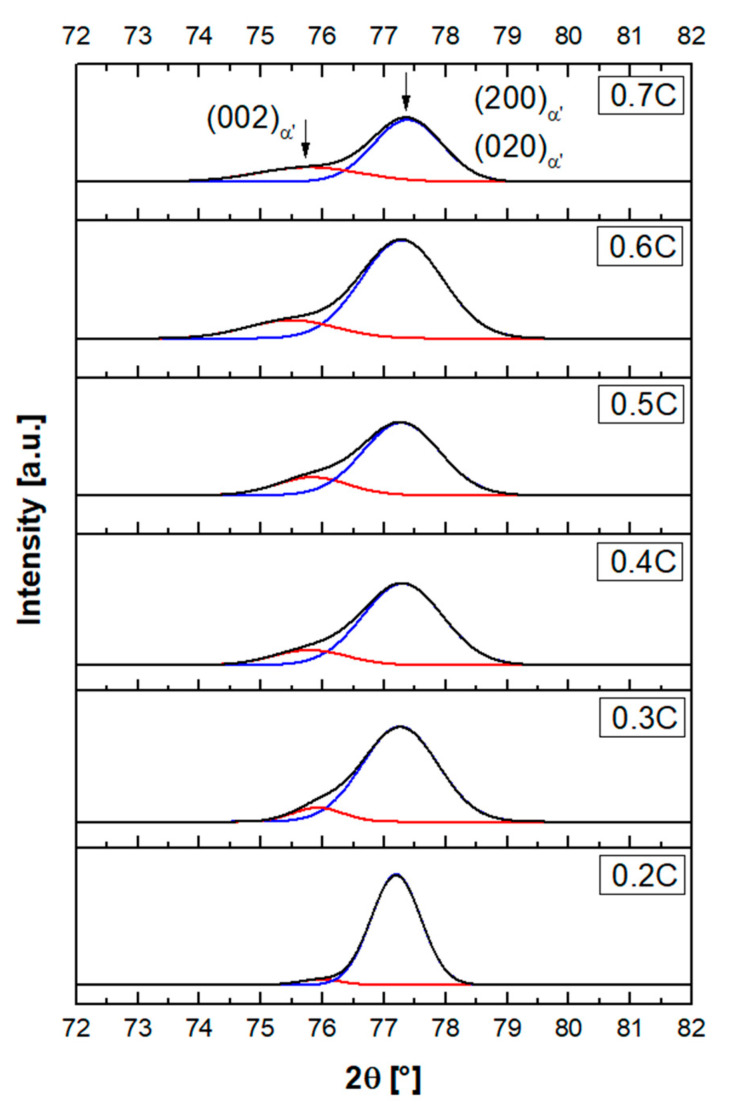
Martensite {002}_α′_ peak profile in the specimens obtained after quenching to 20 °C (black). Narrow lines in red and blue are the deconvoluted Gaussian fits.

By Rietveld refinement of XRD patterns, the lattice parameter of constituent phases in alloys, after quenching to RT, was determined. At high carbon concentrations, the martensite peaks indicated clear tetragonality of the unit cell. This is demonstrated by diffraction patterns in the range 2θ = 72–82°, which covers the occurrence range of {002}_α′_ peaks (Figure 10). As the carbon concentration increases, the relative intensity of the (002)_α′_ peak increases at the expense of the (200)_α′_ and (020)_α′_ peaks. Gaussian fits to these two peaks are superimposed in Figure 10. Peak splitting is noticeable at carbon contents equal to or larger than 0.2%, which agrees with the reported minimum carbon level for a detectable martensite tetragonality [77,78].

**Figure 11:**
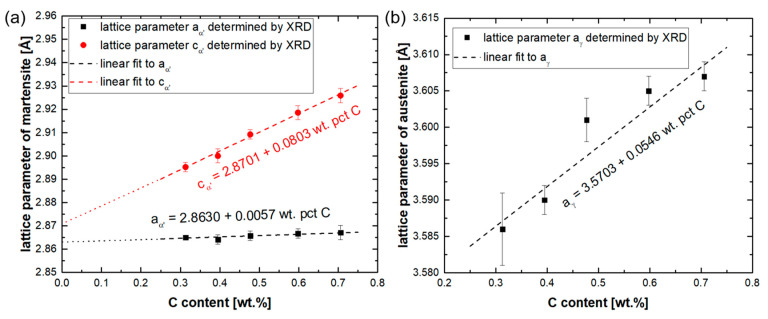
(**a**) Lattice parameters $a_{\alpha^{\prime }}$ and $c_{\alpha^{\prime }}$ of martensite as a function of the carbon content; (**b**) lattice parameter of austenite $a_{\gamma }$ as a function of the carbon content.

The measured lattice parameters of martensite and austenite are shown in Figure 11. By increasing the carbon content, the tetragonality of martensite becomes more pronounced [79]. Due to the low tetragonality of martensite, the lattice parameters $a_{\alpha^{\prime }}$ and $c_{\alpha^{\prime }}$ were not quantified for the 0.2C steel. In contrast to the procedure adopted by Roberts [80] and Lu et al. [81], for carbon and low-alloy steels, no effort was made to enforce the coincidence of extrapolated $a_{\alpha^{\prime }}$ and $c_{\alpha^{\prime }}$ values at a carbon concentration of zero. The results of least squares fitting, based on all the data, yield the following lattice parameters for the martensite and austenite:

(1) $$a_{\alpha^{\prime }}=2.8630+0.0057w_{C}$$

(2) $$c_{\alpha^{\prime }}=2.8701+0.0803w_{C}$$

(3) $$a_{\gamma }=3.5703+0.0546w_{C}$$

where $w_{C}$ refers to the carbon content in wt.%, and $a_{\alpha^{\prime }}$, $c_{\alpha^{\prime }}$ and $a_{\gamma }$ are the lattice parameters in angstrom. The validity of these three equations is limited to carbon contents between 0.313 and 0.706%. In Figure 11b, the deviations in the lattice parameters of austenite in XRD measurements from a linear relationship are very clear. In materials with an fcc crystal structure and a low stacking fault energy, an anisotropic (hkl)-dependent peak shift takes place [82]. In other words, some lattice planes experience an expansion, whereas some others are contracted, which is due to the presence of stacking faults. This causes the XRD peaks for austenite to shift to smaller or larger angles relative to their ideal position in the absence of stacking faults [57], thereby increasing the error in the lattice parameter quantification by full-profile fitting. Therefore, the presence of stacking faults is a likely explanation for the large error associated with the quantification of the austenite lattice parameter in the present steels. 

### 3.6. Hardness of Martensite and Austenite Mixtures

**Figure 12:**
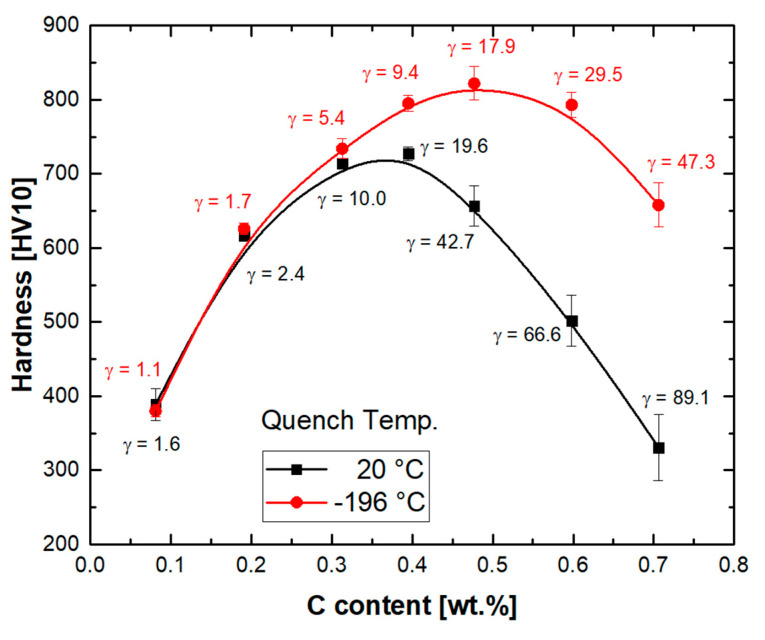
Vickers hardness values vs. carbon content for steels quenched to two different temperatures as marked. Number next to each symbol indicates austenite fraction in vol.%. Except for the 0.1C alloy whose microstructure consisted of martensite, ferrite, and austenite, the rest of alloys had two-phase martensitic–austenitic microstructures.

The hardness values for the specimens quenched to RT and −196 °C (microstructures in Figure 7 and Figure 8) are shown in Figure 12. To obtain values representative of the phase mixtures, measurements were conducted using a load of 10 kg. This ensured indentation sizes large enough compared to the size of the microstructure constituents. The hardness values for the specimens quenched to RT increase from 389 HV10 for the 0.1C alloy to 728 HV10 for the 0.4C alloy. The hardness increase by alloying with carbon can be attributed to the solid solution strengthening of martensite. The significant solid solution strengthening of martensite by carbon can be explained by the lattice distortions induced due to the increased occupancy of interstitial lattice sites [83]. Increased tetragonality, in turn, opposes the dislocations glide and enhances the strength. 

As the carbon concentration exceeds 0.4%, the hardness starts to decrease. The decrease in hardness occurs in spite of further increase in the lattice microstrains in martensite, as evidenced by the increased tetragonality of martensite (Figure 11a). A similar decrease in hardness occurs for as-quenched carbon steels at carbon concentrations above 0.9%. This is known to be related to the presence of retained austenite [84]. In the present case, the decrease in hardness begins as the retained austenite content exceeds 20 vol.%. Not only the fraction of retained austenite, but also its mechanical stability, are decisive to the hardness of steels with martensitic–austenitic microstructures [13,85]. The observation of a hardness of only 330 HV10 for the 0.7C steel consisting of 89.1 vol.% austenite indicates that the mere solid solution strengthening of austenite by carbon is much weaker than that of martensite. In practice, an increase in the carbon concentration of austenite is expected to lower its strength further, by raising its stacking fault energy [86], and, thereby, its mechanical stability [87]. The deformation-induced martensite formation as an effective strain hardening mechanism is then delayed, resulting in a decrease in hardness [88]. The ready transformation of austenite with a low mechanical stability, on the other hand, enhances the strength. Phase-specific nano-indentation measurements of a Q&P steel containing austenite with a low stability against deformation-induced martensite formation have indicated comparable hardness values for both martensite and austenite constituents [89].

For the steels quenched to −196 °C, the variation in hardness with carbon content resembles those of specimens quenched to RT. Nevertheless, due to the increase in the fraction of martensite, the hardness values are somewhat higher. The increase in hardness due to cryogenic cooling is especially pronounced for steels with higher carbon concentrations, as they experience a larger increase in the martensite fraction. Therefore, the critical carbon concentration at which the hardness maximum is reached shifts to a higher carbon concentration (0.5C). Due to the expected partitioning of both stress and strain between martensite and austenite [90], neither linear nor inverse rules of mixtures are applicable for the estimation of the hardness of individual phases as a function of the carbon concentration [91].

A comparison of the hardness values in Figure 12 to those for as-quenched carbon steels [84] indicates that at a given carbon concentration, martensite in the present stainless steels has a higher hardness. The solid solution strengthening of Cr, and a possible synergy with carbon, might have played a role [14,21]. The observation of maximum hardness at a lower carbon concentration, on the other hand, can be attributed to the reduction in M_s_ temperature by Cr [45]. The critical austenite fraction required to lower the hardness of the phase mixture is then reached at a lower carbon content.

## 4. Conclusions

The influence of carbon content on the microstructure and mechanical properties of stainless steels with the base chemical composition Fe–13Cr (wt.%) and carbon concentrations between 0 and 0.7 wt.% was studied between −196 °C and the liquid range. The following conclusions were drawn:

1. By DSC measurements, the solidification mode was found to change from ferritic to ferritic–austenitic as the carbon concentration increased. The experimental results were in reasonable agreement with the thermodynamic calculations. The solidus–liquidus interval was, however, displaced and broader in the DSC measurements.
2. Based on the DSC results, aided by microstructural examinations in the as-quenched state, alloys containing nearly 0% C and 0.1%C exhibited fully ferritic and austenitic–ferritic high-temperature microstructures, respectively. Alloys containing 0.2–0.7%C, on the other hand, exhibited a fully austenitic phase field.
3. Quenching to RT after heat treatment of alloys in the austenite range resulted in the partial transformation of austenite to athermal martensite. The fraction of retained austenite increased with the carbon concentration. This was due to the reduction in the martensite start temperature, by about 629 °C per wt.%C. The phase fraction of retained austenite was reduced by cooling to −196 °C.
4. According to the XRD results, the tetragonality of the martensite unit cell becomes detectable at a carbon concentration of 0.2%, and continuously increases at higher carbon concentrations. The variations in lattice parameters with carbon concentration for martensite ($a_{\alpha^{\prime }}$ and $c_{\alpha^{\prime }}$) and austenite ($a_{\gamma }$) were expressed by linear equations in the following form: $a_{\alpha^{\prime }}=2.8630+0.0057w_{C}$, $c_{\alpha^{\prime }}=2.8701+0.0803w_{C}$ and $a_{\gamma }=3.5703+0.0546w_{C}$, with $w_{C}$ denoting carbon concentration in wt.% and lattice parameters expressed in angstrom. The equations are valid for 13%Cr steels with carbon contents between 0.313 and 0.706 wt.%.
5. The variation in hardness with carbon concentration for steels with martensitic–austenitic microstructures indicated a maximum at intermediate carbon concentrations. Given the steady increase in the tetragonality of the martensitic constituent at higher carbon concentrations, as confirmed by the X-ray diffraction measurements, the observed variation in hardness with carbon concentration was justified by the amount and stability of the coexisting austenite.

## Figures and Tables

**Table 1:** Chemical composition of alloys in wt.%.

Alloy ID	C	Cr	Fe + Trace Elements
0C	0.004	13.7	balance
0.1C	0.081	13.1	balance
0.2C	0.191	12.9	balance
0.3C	0.313	13.1	balance
0.4C	0.395	13.3	balance
0.5C	0.477	13.1	balance
0.6C	0.598	12.8	balance
0.7C	0.706	13.1	balance

**Table 2:** Overview of alloy-specific austenitization conditions.

Alloy ID	Austenitization Temperature (°C)	Holding Time (min)
0.1C	1100	10
0.2C	1100	10
0.3C	1150	10
0.4C	1150	10
0.5C	1200	10
0.6C	1300	5
0.7C	1300	5

## Data Availability

The raw/processed data required to reproduce these findings cannot be shared at this time as the data also forms part of an ongoing study.
